# Estrogenized HSA induced high-affinity autoantibodies in breast cancer - Novel biomarker for early detection

**DOI:** 10.3389/fonc.2024.1493320

**Published:** 2024-11-27

**Authors:** Subuhi Sherwani, Mohd Wajid Ali Khan, Wahid Ali Khan, Saravanan Rajendrasozhan, Khalid Al-Motair, Hamda Khan, Saheem Ahmad

**Affiliations:** ^1^ Department of Biology, College of Science, University of Hail, Hail, Saudi Arabia; ^2^ Medical and Diagnostic Research Center, University of Hail, Hail, Saudi Arabia; ^3^ Department of Chemistry, College of Science, University of Hail, Hail, Saudi Arabia; ^4^ Department of Clinical Biochemistry, College of Medicine, King Khalid University, Abha, Saudi Arabia; ^5^ Department of Biochemistry, J.N. Medical College, Aligarh Muslim University, Aligarh, India; ^6^ IIRC-1, Department of Biosciences, Integral University, Lucknow, India; ^7^ Department of Medical Laboratories, College of Applied Medical Sciences, University of Hail, Hail, Saudi Arabia

**Keywords:** estrogen, HSA, breast cancer, ELISA, autoantibodies, molecular target, BC biomarker

## Abstract

**Objective:**

Breast cancer (BC) is the second most prevalent cancer worldwide. Estrogen has been increasingly recognized as a major contributor to the development of BC, playing a more critical role than previously understood. Estrogen derived nucleic acid and protein adducts have been shown to play significant roles in BC development and progression. However, the alterations in molecular mechanism(s) and immune pathways arising as a result of estrogenization still remain elusive.

**Patients and methods:**

4-hydroxyestradiol (4-OHE_2_) was used for adduct formation with protein human serum albumin (HSA) (4-OHE_2_-HSA). The affinity of antibodies for 4-OHE_2_-HSA was evaluated in breast cancer patients. Immunoassays (direct binding ELISA, inhibition ELISA, and quantitative precipitin titration assay) were used to assess autoantibodies against estrogenized HSA in BC patients (n = 85) and healthy controls (n = 45).

**Results:**

Estrogenization of HSA altered both its structure and function and compromised its interactions with various HSA-binding proteins. BC patients demonstrated high-affinity antibodies against 4-OHE_2_-HSA as compared to HSA (*p* < 0.05). Additionally, cytokines Interleukin (IL)-1, IL-6 and tumor necrosis factor-alpha (TNF-α) were significantly elevated in BC patients as compared to the control group. Several factors, such as chemotherapy, estrogen receptors (ERs), and combination of surgery and chemotherapy, influenced the production of antibodies in cancer patients. The affinity constant for estrogenized HSA was 1.31 × 10^-7^ M, while for HSA and 4-OHE_2_, it was 1.68 × 10^-6^ M and 1.36 × 10^-6^ M, respectively.

**Conclusions:**

Estrogenized HSA is highly immunogenic, resulting in functional alterations. High affinity antibodies were detected in BC patients against 4-OHE_2_-HSA. Consequently, 4-OHE_2_-HSA may serve as a novel molecular target for potential cancer therapeutics. Furthermore, autoantibodies against 4-OHE_2_-HSA could serve as a potential biomarker for early detection of BC.

## Introduction

Breast cancer is among the most common and fatal cancers in women, representing a significant proportion of cancer-related deaths globally (1, 2). In 2022, approximately 2.296 million new cases of BC were diagnosed globally in women, with an estimated 666,103 related deaths. The highest number of new cases (357,161) occurred in China (3). Additionally, France reported the highest age-standardized incidence rate of BC in 2022, at 105.4 per 100,000 women (3). In terms of mortality, India recorded the highest number of BC-related deaths, with 98,337 deaths reported during the same year (3). The disease, along with its associated complications and treatments, frequently imposes substantial physical and psychological burdens on patients. Early diagnosis, effective monitoring, and precise treatment are key to improving prognosis and quality of life (4, 5).

Various factors have been implicated in the progression of breast cancer, with lifetime exposure to estrogen being a significant contributor (6). Estradiol can influence disease progression through its interaction with estrogen receptor (ER) (6). Estrogen receptor expression in BC plays an important role in carcinogenesis and disease advancement (7). BC patients exhibit adducts of serum protein in their blood (8), including reported adducts formed between estrogen and immunoglobulin (IgG) (9). In general, proteins undergo covalent modification by catechol estrogens (CEs) (2-hydroxyestradiol or 4-hydroxyestradiol) (10), in a process referred to as ‘estrogenization of protein’. Insulin is another protein that undergoes estrogenization and is relevant in the context of type I diabetes (10). Additionally, the binding of estrogen to different proteins and adducts thus formed, may be associated with prostate cancer, endometrial cancer, type I diabetes, and other malignancies (11–13).

The level of estrogen is an important factor in the development of breast cancer (14). Estrogen-related cancers have been linked to a receptor-mediated mechanism (15). Additionally, the carcinogenic process can also occurs through damage to DNA caused by catechol estrogens (16, 17). Reactive oxygen species (ROS) produced by CEs can modify DNA and produce autoantibodies in BC (17). Cytochrome P_450_ catalyzes the oxidation of estradiol to catechol estrogen, such as 2-OHE_2_ and 4-OHE_2_ (18). Further metabolism of CEs produces reactive estrogen quinone such as estradiol-2,3-quinone and estradiol-3,4-quinone (19). These CEs can convert to quinone and semi-quinone forms, subsequently forming adducts with DNA bases (16). The levels of CEs in the serum reflect estrogen homeostasis, and their concentration may vary in blood. However, CEs conjugated proteins (estrogenized proteins) are stable in blood and serve as a more reliable biomarker compared to CEs alone.

Albumin and hemoglobin adduct of estrogen-3,4-quinone and estrogen-2,3-quinone may serve as biomarkers for early detection of breast cancer (8). The use of radiolabeled HSA nanoparticles combined with methotrexate and transtuzumab has been reported (20) in breast cancer diagnosis and treatment. HSA nanoparticles can also function as carriers for targeted delivery of antibiotics to manage bacterial infections (21). Low serum albumin and elevated neutrophil counts are correlated with poor prognosis for metastatic breast cancer (22). Flexible HSA nano-capsules enhance drug delivery and cellular uptake for chemocancer-based therapy (23). HSA nanoparticles with 7-ethyl-10-hydroxyl camptothecin could be a promising strategy for breast cancer chemotherapy (24).

In this study we have assessed the affinity of antibodies against estrogenized human serum albumin, given that estrogenized proteins are frequently observed in cancer patients, and HSA which is the most abundantly found protein in blood. Consequently, estrogenized HSA may function as a significant biomarker for BC detection. To investigate the possible role of estrogenized HSA in BC, estrogenized HSA was screened in the serum of BC patients, and the possible role of 4-OHE_2_-HSA was assessed.

## Patients and methods

### Patients and controls

The study consists of two groups of female subjects. Group one included 85 patients presenting with clinical signs and symptoms of breast cancer. Group two consisted of 45 healthy individuals who served as controls. All samples were collected following the acquisition of informed written consent from participants. This study has been approved by the university institutional ethics committee, with further details provided in the ‘Ethics Statement’ section.

The statistical power of the experimental group sample size was calculated to be 0.996 at a significance level 0.05. Inclusion criteria comprised women aged 18-90 years, clinical signs and symptoms of breast cancer, and a clinical diagnosis of breast cancer confirmed with histopathology and other symptoms. Aseptic techniques were employed for the collection of all the samples. Both patients and controls underwent similar diagnostic procedures, with patients clearly separated from the control group. Exclusion criteria included pregnancy or lactation, patients on tamoxifen therapy, alcohol consumption, or thyroid medication. Control group consisted of women who were blood donors. **Table 1** presents data of BC patients and controls. The average age of the patients was 61.4 ± 9.2 years. Among the cancer patients, 61 (71%) were estrogen receptor positive, while 24 (28%) were negative, with receptor status determined by using immunohistochemistry, based on more than 10% of stained cells. Among the patients, 29 (34%) were premenopausal and 56 (65%) had reached menopause. 40% (34) of the patients were classified as Grade 1 (characterized by cancer cells that resemble normal cells with a slow growth rate), 48.2% (41) as Grade 2 (cancer cells with abnormal morphology and faster growth rate than normal cells), and 11.8% (10) as Grade 3 (characterized by cells with highly abnormal morphology and rapid growth rate with potential to spread to other tissues or organs). The average body mass index of the patients was 26.9, indicating they were slightly overweight. Additionally, 24.6% of cancer patients reported a family history of breast cancer, and 45 identified as smokers. In terms of treatment among the patient group, there were 23 women in the chemotherapy group, 15 in the surgery group, 10 in the surgery + radiotherapy group, 17 in the surgery + chemotherapy group, and 20 had not yet started any therapy or treatment. All the patients in the chemotherapy group took cyclophosphamide, methotrexate, and 5-fluorouracil. The majority (85%) were married and had completed high school (95%).

**Table 1:** Clinical data of breast cancer patients and normal healthy individuals as controls.

Characteristics	Breast cancer (n=85)	Controls (n=45)
Age (years)	61.4±9.2	60.8±6.9
Disease duration (yrs)	9.3±5.8	_
Estrogen receptor		
Positive (n)	61 (71%)	_
Negative (n)	24 (28%)	_
Menopausal status		
Premenopausal (n)	29 (34%)	_
Postmenopausal (n)	56 (65%)	_
Grading		
Grade 1	34 (40%)	_
Grade 2	41 (48.2%)	_
Grade 3	10 (11.8%)	_
Smoking		
Current/Past(n)	45	18
Never (n)	40	27
Inflammatory cytokines estimation^*^		
IL-6	33.42±6.42***	8.92±1.58
IL-1	97.67±18.36*	62.90±13.33
TNF-α	102.15±21.51**	39.91±8.13

^***^ p<0.001,^**^ p<0.01, ^*^ p<0.05. Cytokines were estimated in pg/ml.

n, number of subjects; IL-6, interleukin-6; IL-1, interleukin-1; TNF-α, tumor necrosis factor-alpha.

**Figure 1:**
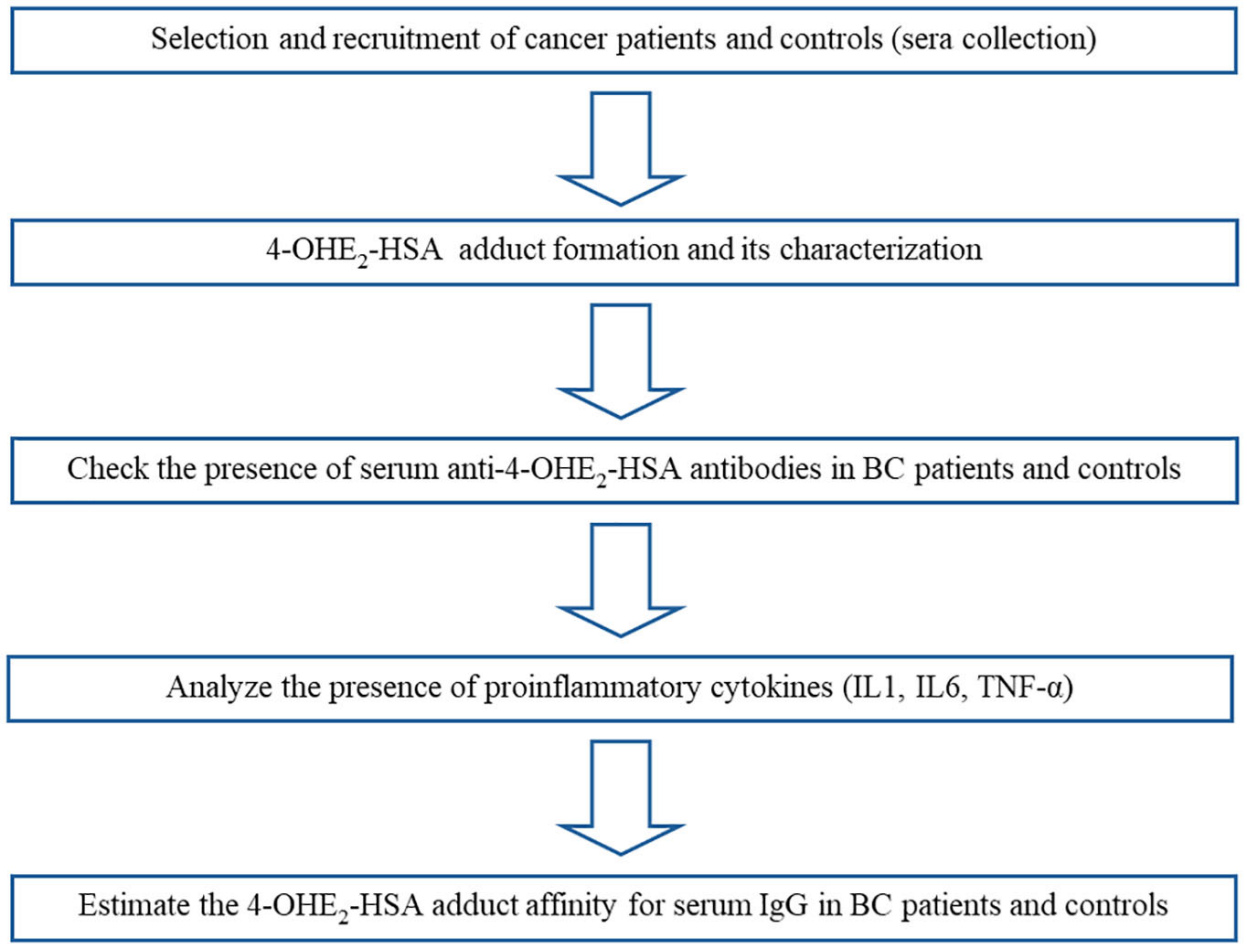
Illustration of the workflow of the study.

Serum samples were collected and put in a water bath at 56°C for 30 min to inactivate complement proteins. All serum samples were stored at -20°C, and 0.1% sodium azide was added to the samples as a preservative before storage. Multiple aliquots were prepared for each sample to be used in different experiments and stored at -20°C. One aliquot was used for one experiment and then discarded. The workflow is illustrated in **Figure 1**, and the step-by-step procedure has been explained in subsections.

### Materials

HSA, 4-OHE_2_, N,N-dimethylformamide (DMF), Cubilin, Megalin and Fc receptor (FcRn), Fetal calf serum (FCS), sodium pyruvate, L-glutamate, 3-[4,5-dimethylthiazol-2-yl]-2,5-diphenyltratrazolium bromide (MTT), RPMI medium, dimethyl sulfoxide (DMSO), anti-human IgG-alkaline phosphate conjugate, p-nitrophenyl phosphate, sodium chloride (NaCl), and 3, 3’, 5, 5’-tetramethylbenzidine (TMB) were purchased from Sigma-Aldrich, St. Louis, MO, USA. Human interleukin (IL)-6 and IL-1 ELISA Kit (Sigma-Aldrich, Chemie GmbH, Darmstadt, Germany). anti-Cubilin antibody-horse reddish peroxidase (HRP), anti-Megalin antibody-HRP, anti-FcRn antibody-HRP, and anti-human IgG antibody-HRP were purchased form (Santa Cruz). TNF-α High sensitivity ELISA Kit (IBL International GmbH, Hamburg, Germany). MCF-7 cell lines were procured from the American Type Culture Collection in Manassas, VA, USA.

### Estrogenized HSA adduct formation

The procedure for the estrogenization of HSA was followed according to Ku et al. (25) with slight modifications. Varied concentrations of 4-hydroxyestradiol (4-OHE_2_) were used (1-15 mM) for the modification of HSA (1-5 mg/ml). After standardization of the procedure, 4-hydroxyestradiol (4-OHE_2_) (11 mM) was incubated with 1 mg/ml of HSA in 20 mM phosphate buffer (pH = 7.4), at 37°C for 6 h. 4-OHE_2_ was solubilized in N,N-dimethylformamide (DMF) at a concentration not exceeding 0.1%. Any extra 4-OHE_2_, which was not bound in the process of estrogenization, was finally removed by dialyzing the sample in 20 mM PBS (pH = 7.4) (12).

### UV-Vis Spectral analysis

UV-Vis spectrophotometry (Shimadzu spectrophotometer-model UV-1700; Kyoto, Japan) was employed to study HSA and estrogen-modified HSA in the range of 200-400 nm. Sample concentrations for native and modified samples were 150 µg/ml and quartz cuvette with 1 cm path length analyzed the samples. The peaks were observed at 280 nm as in published methods (10, 26).

Change in chromicity in HSA molecules were assessed by using the formula:


$$\frac{\text{Native HSA Absorbance}-\text{Modified HSA Absorbance}}{\text{Native HSA Absorbance}}\times 100$$

### Fluorescence spectroscopy analysis

Hitachi model F2000 spectrofluorometer (Japan) was used to obtain the fluorescence spectrum of native HSA and 4-OHE_2_-HSA solutions were recorded. The HSA concentration for both the samples were 60 µM. Emission spectra with a wavelength range of 290–430 nm were obtained using excitation wavelength of 280 nm with scanning speed of 1000 nm/min. Fixed slit widths used were 5 nm. PBS 20 mM buffer solution was used as a blank.

### Cancer cell lines

Breast cancer cell line (MCF-7 cells) was grown in complete Rosewell Park Memorial Institute (RPMI) 1640 medium using supplements [nonessential amino acids, 5% Fetal calf serum (FCS), sodium pyruvate (1 mM), and L-glutamate (2 mM)]. Antibiotic penicillin/streptomycin (50 μg/ml) (GIBCO Life Technologies, Carlsbad, CA, USA) was also added to the RPMI medium. Cell culture was maintained at 37°C in a carbon dioxide (5%) incubator.

### MTT assay

The toxicity of antigens (native and modified) on the breast cancer cells was analyzed using the MCF-7 cell line. A well-known 3-[4,5-dimethylthiazol-2-yl]-2,5-diphenyltratrazolium bromide (MTT) assay was used, as reported earlier (27). MCF cells (1 × 10^4^ cells/mL) were taken in 96 well plates in a complete RPMI medium. Varying concentrations of the antigens (4-OHE_2_-HSA, HSA, 4-OHE_2_) (0-1 4 µg/ml) were incubated with MCF-7 cells for 24 h at 37°C in a carbon dioxide (5%) incubator. After 24 h incubation, 100 μl of MTT (5 mg/ml) was added and kept at 37°C for 4 h. Medium was aspirated from wells including MTT solution. A 50 μl DMSO solution was added to remaining formazan crystals, and results were recorded at 570 nm. Untreated cells were used as negative control, and cytotoxicity index was estimated. Percent cytotoxicity was calculated as given in the equation:


$$\%\text{ cytotoxicity}=\frac{\left[1-\text{absorbance of experimental well}\right]}{\text{absorbance of negative control well}}\times 100$$

### ELISA

Antibodies from cancer patient sera were checked by direct binding ELISA (10–13). The specific binding of cancer antibodies with estrogenized HSA was also detected by inhibition ELISA (10–13). Briefly, in inhibition ELISA, 100 μl of estrogenized HSA, with a concentration of 2.5 μg/ml, was used to coat microtiter plates. The mixture was kept for two h at room temperature and later at 4°C overnight. These were washed with tris-buffer saline and tween [TBS-T; T (20 m M Tris, 2.68 m M KCl, 150 m M NaCl, pH 7.4, containing 0.05% Tween-20)] (TBS-T) and then blocked with 1.5% bovine serum albumin (BSA) in TBS buffer (20 m M Tris, 2.68 m M KCl, 150 m M NaCl, pH 7.4). Immune complexes with different antigens were formed by taking 100 μl of 1:100 dilution of cancer sera with different amounts of antigens (estrogenized HSA, HSA, 4-OHE_2_). Similarly, immune complexes were prepared taking 100 μl of IgG (1 μ/ml) BC patients and healthy individuals with different amounts of antigens (estrogenized HSA, HSA, 4-OHE_2_). The complexes were initially kept for 2 h at 37°C and then at 4°C for 12 h. In each well of the microtiter plates, immune complexes (100 µl) were added, followed by anti-human IgG-alkaline phosphatase conjugate. P-nitrophenyl phosphate was used to develop the reaction, and finally, reading was recorded at 410 nm using a microplate reader (MR9600-415 Accuris, NJ, United States).

Estimations for cytokines IL-6, IL-1 and TNF-α were made from serum samples of the cohorts using quantitative sandwich ELISA based on commercially available ELISA kits. According to the kit protocol the cytokines were detected with a sensitivity of < 0.5 pg/ml. Each sample was run in duplicate.

### Anti-4-OHE_2_-HSA antibodies – isolation and purification

A Protein A-Agarose column was used to isolate and purify IgG from serum samples. IgGs were isolated from cancer patients using an affinity chromatography technique (10). Sera samples were diluted with PBD (20 mM, pH 7.4) in a ratio of 1:1. Protein A-Agarose column was washed (3x) with the same PBS buffer and then diluted samples were loaded to the column and eluate was collected and reloaded on the column. This was repeated 2-3 times to ensure excessive binding of the IgG present. Column was washed with PBS (20 mM, pH 7.4) to remove unbound IgGs. Acetic acid 0.6 ml was prepared using 0.85% NaCl. This solution was neutralized with 1 mL Tris-HCl (1 M, ph 8.5). Eluted samples were collected in a volume of 3 ml each and analyzed on spectrophotometer at 251 and 278 nm wavelengths. Estimation of isolated IgG was done using the formula 1.40 OD_280_ = 1.0 mg/ml. Isolated IgGs from samples were stored at -20 C with 0.1% sodium azide.

### Binding of estrogenized HSA or HSA alone with cubilin, megalin, FcRn, and patients’ IgG

HSA undergoes structural alteration due to estrogenization which may cause loss of functional activity. This can be analyzed by direct binding ELISA, in which the interactions of 4-OHE_2_-HSA or HSA alone with HSA binding proteins (Cubilin, Megalin), FcRn, and IgG from BC patients (n=3) were assessed as given in previously published method (10–13). Briefly, polystyrene 96 well-microplates were coated with 100 μl of 4-OHE_2_-HSA or HSA (2.5 μg/ml) and incubated for 3-4 hours at room temperature. After incubation, the microplates were washed thrice with TBS-T and then blocked with 1.5% skimmed milk in TBS buffer. These were incubated for 4 hours and then the microplates were washed 3-5 times with TBS-T. Subsequently Cubilin, Megalin, FcRn, and patients’ IgG (n=3) were added to separate plates and incubated for 2 hours at room temperature in the dark and later kept at 4°C overnight for maximum interaction of native or modified HSA with proteins. Next day these plates were washed thrice with TBS-T. Then 100 µl (0.05 µg/ml) of anti-Cubilin antibody conjugated horse reddish peroxidase (HRP), anti-Megalin antibody-HRP, anti-FcRn antibody-HRP, and anti-human IgG antibody-HRP were added to the respective plates and kept for 2 hours at room temperature. Secondary antibodies were washed with TBS-T (3 times) and substrate 3, 3’, 5, 5’-tetramethylbenzidine (TMB) was added to all the plates and incubated for 10-15 minutes for color development. The enzymatic reaction was stopped by adding sulfuric acid (0.18 M) and color was read as optical density at 450 nm using a microplate reader (MR9600-415 Accuris, NJ, United States).

### Estimation of antibodies affinity from patients

IgG from BC patients was tested for their affinity to 4-OHE_2_-HSA by estimating immune complexes formed between IgG from cancer patients and estrogenized HSA (13). In this protocol, a 500 μl reaction mixture consisted of a fixed IgG (100 μg) concentration with varying amounts of estrogenized HSA (1-40 μg). After incubation of the reaction mixtures for 2 h at 37°C, the reaction mixtures were kept in a fridge (4°C) overnight. Immune complexes were centrifuged and pelleted and solubilized in 1N NaCl (250 μl). The colorimetric method was used to determine free or bound protein (28). IgG affinity of cancer patients was thus determined (13).

### Statistical analysis

Statistical differences between the controls and BC patients were calculated using the Student’s *t*-test and ANOVA analysis with GraphPad Prism 4.0 software for Windows 10 (Boston, MA, USA). Significance was considered if the value *p* < 0.05. Data presented as mean ± SD values and samples in each assay were run in triplicate.

## Results

### 4-OHE_2_-HSA: formation and characterization

**Figure 2:**
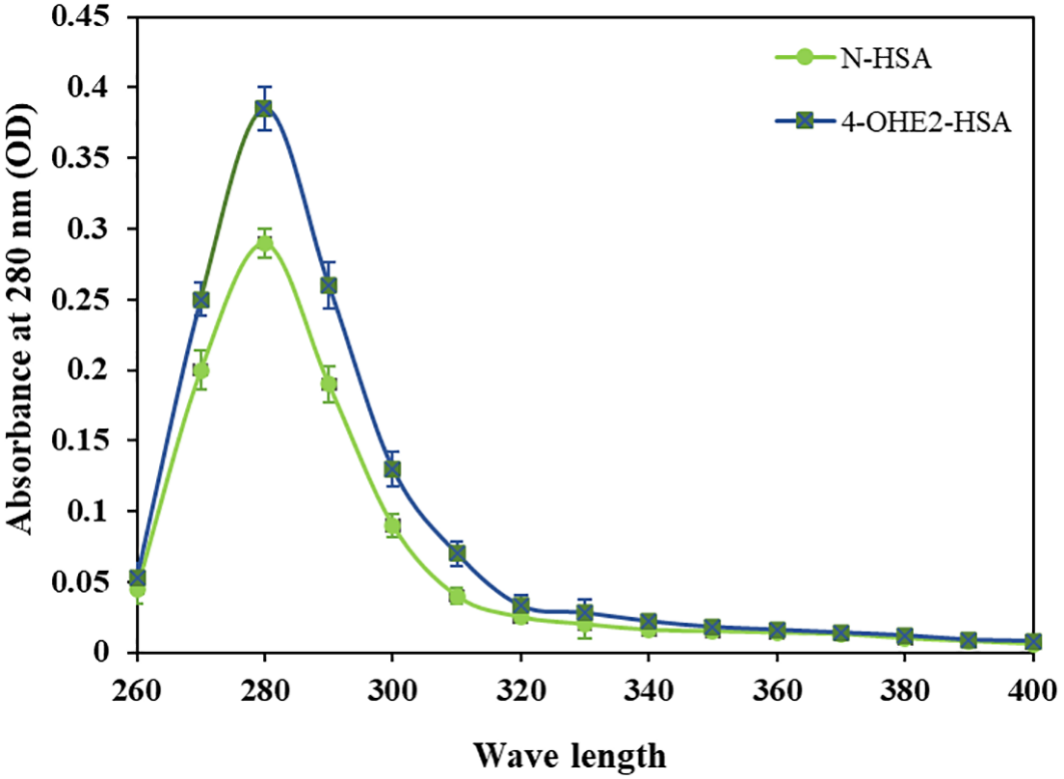
Ultraviolet absorption spectra of HSA (green) and 4-OHE_2_-HSA (blue) adduct were recorded at 280 nm. OD represents optical density. Data presented as mean ± SD values.

**Figure 3:**
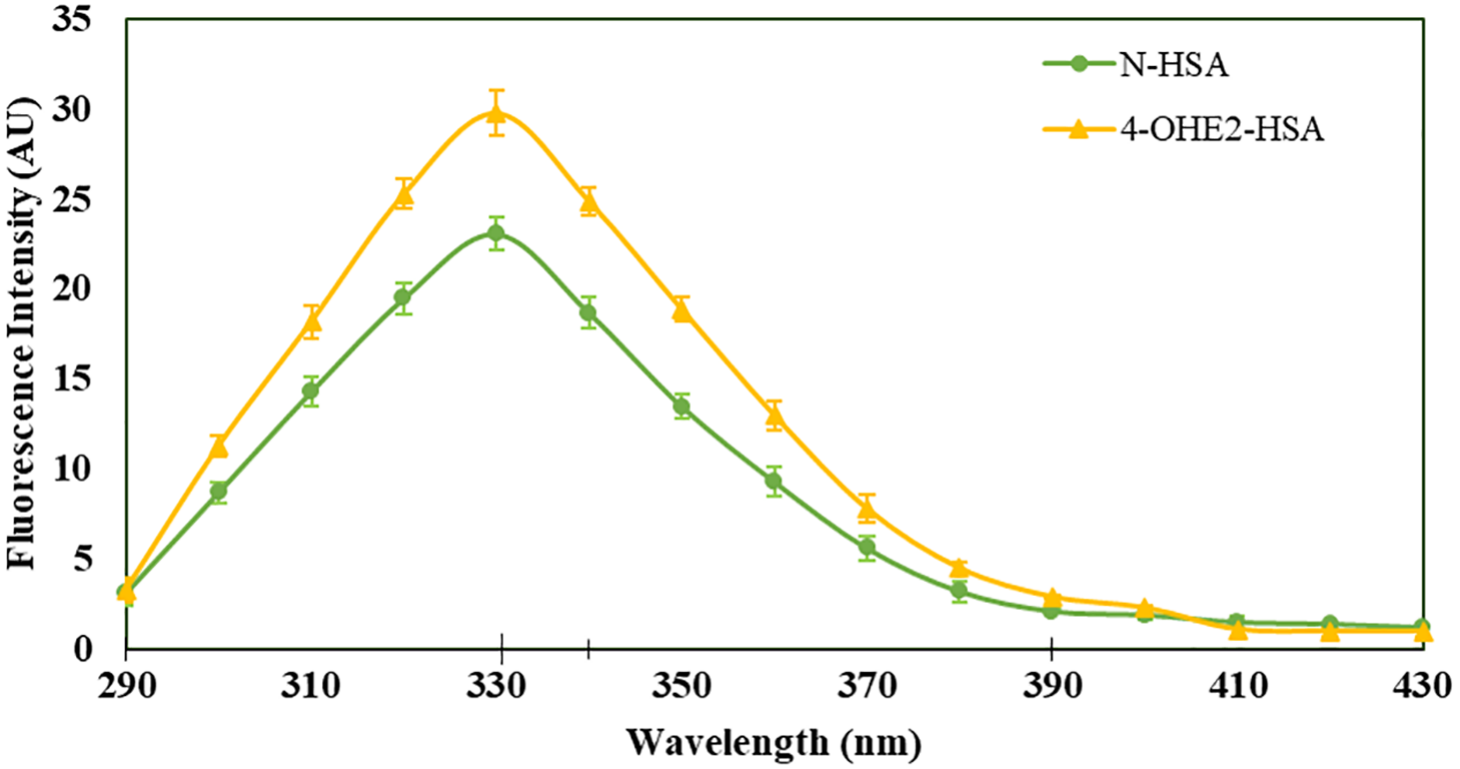
Tryptophan specific fluorescence intensities were estimated for HSA (green) and 4-OHE_2_-HSA (yellow). Excitation of samples at 285 nm and emissions recorded at 330 nm for HSA and at 310 nm for 4-OHE_2_-HSA. Data presented as mean ± SD values.

Incubation of 4-OHE_2_ with HSA exhibited an increase in UV intensity, as shown in **Figure 2**. The HSA-4-OHE_2_ complex demonstrated UV hyperchromicity of about 33% compared to native HSA. Tryptophan specific fluorescence also showed an increase in fluorescence intensity by 29.0% (**Figure 3**). Notably, both UV- and tryptophan fluorescence spectral studies showed HSA structural perturbation when adduct was formed with 4-OHE_2._ Binding of 4-OHE_2_ occurs on Lysine, Cystine, and Histidine amino acid residues (9). A molecule of HSA has 59 Lysine, 35 Cystine, and 16 Histidine residues. Some of these may bind with 4-OHE_2_ leading to conformational or structural alterations in albumin.

### Reduced interaction of HSA binding proteins with estrogenized HSA

**Figure 4:**
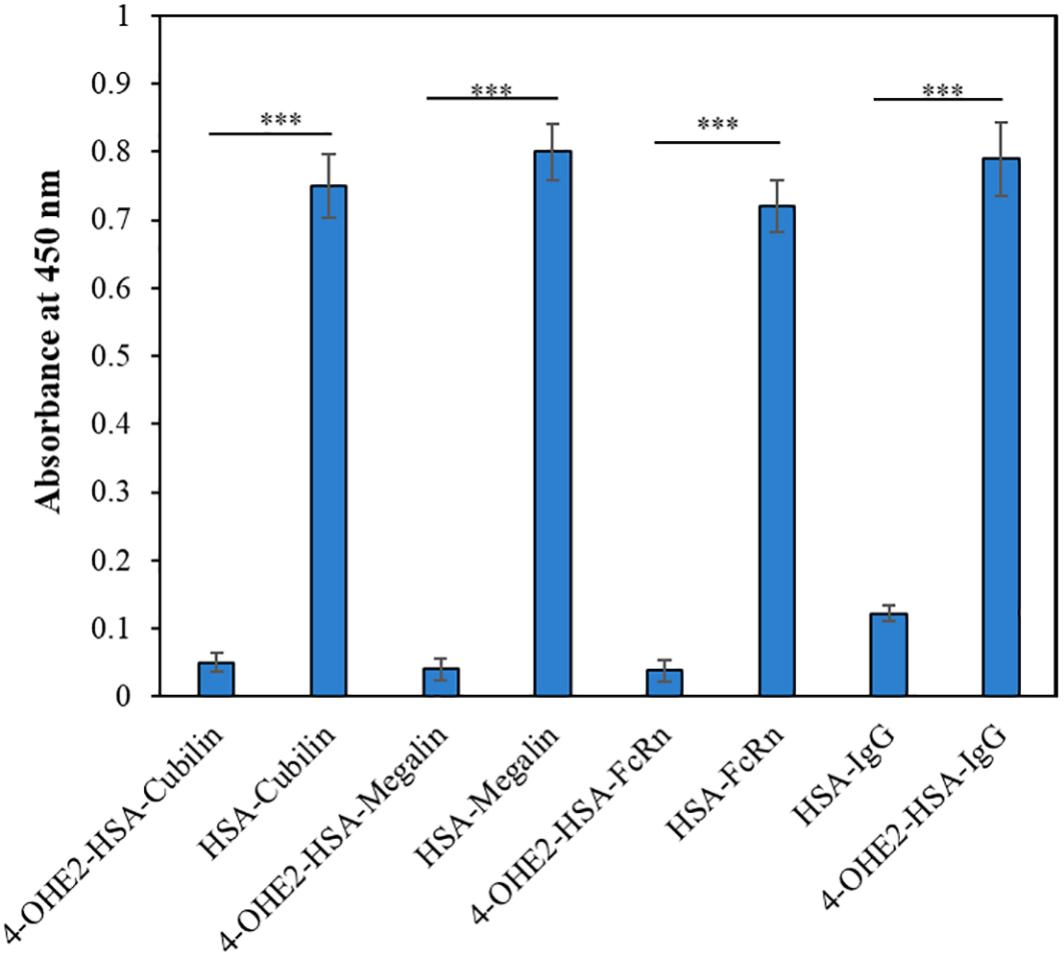
The direct binding ELISA method was employed to analyze the binding of 4-OHE_2_-HSA or HSA with Cubilin, Megalin, FcRn, and BC patients IgG (n=3). Each sample was run in triplicates. Data presented as mean ± SD values. Symbol *** define significance as p < 0.001.

To investigate effects of estrogenization of HSA on the interaction of HSA with various HSA binding proteins (cubilin, megalin) including FcRn, and IgG, the binding was determined by direct binding ELISA, and results were presented as percent binding in **Figure 4**. Estrogenized HSA demonstrated no or minimal binding with various HSA binding proteins, but native HSA showed a high degree of binding with cubilin, megalin, and FcRn. In addition, estrogenized HSA showed a higher affinity towards circulatory IgGs in BC patients, compared to native HSA.

### Cytotoxic effect of 4-OHE_2_-HSA on cell lines

**Figure 5:**
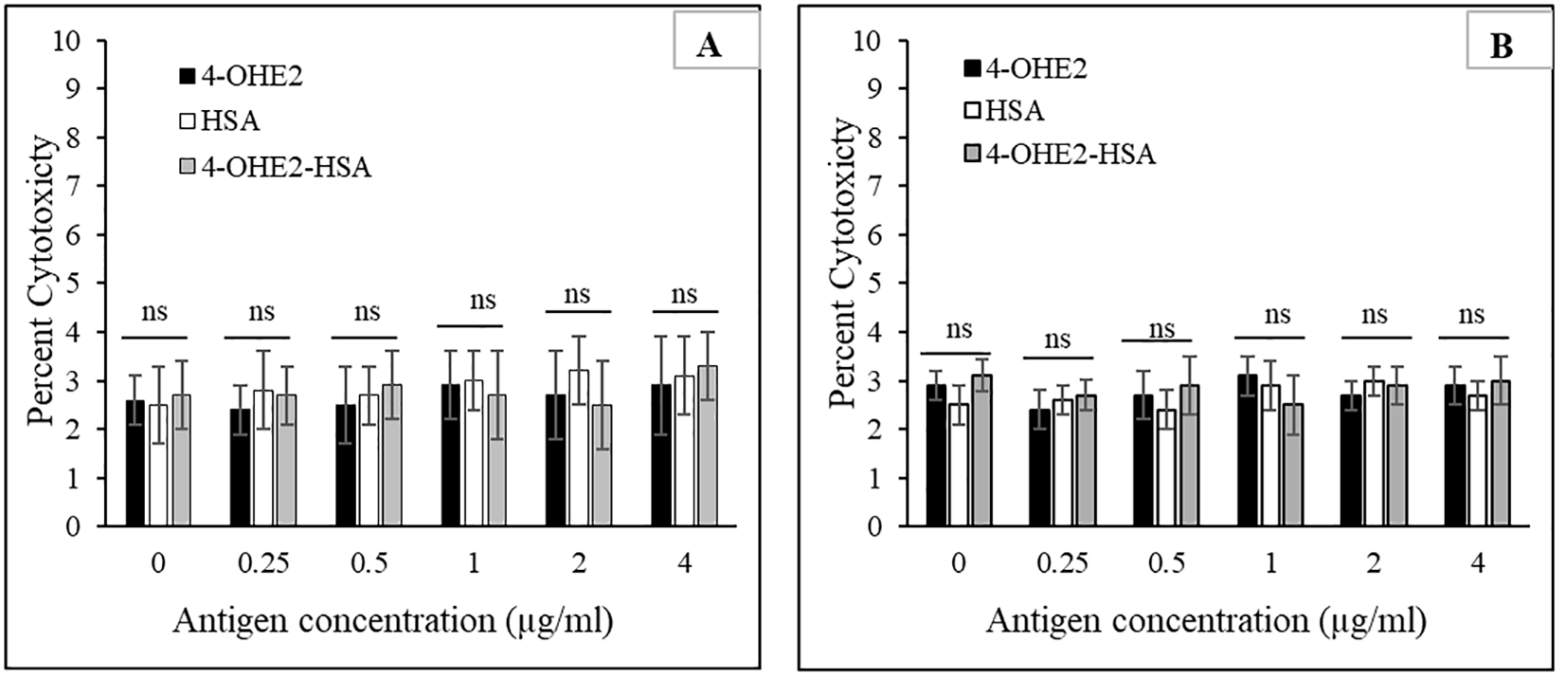
The effect of estrogenized-HSA (4-OHE_2_-HSA) on breast cancer cells MCF-7 **(A)** and PBMC **(B)** was evaluated using an MTT assay. Cancer cells (1 × 10^4^ cells/mL) were incubated with varying concentrations of estrogenized-HSA (0-4 µg/ml) in a 96-well plate. The cell culture was incubated for 24 h at 37°C in a CO_2_ (5%) incubator. 4-OHE_2_ and HSA alone served as controls. Symbol ‘ns’ represents values are non-significant.

Increasing concentrations of native and modified antigens were used to assess their effects on MCF-7 cancer cell proliferation as well as on normal human peripheral blood mononuclear cells (PBMCs). MTT assay was used to measure metabolic activity, and the samples were analyzed at 570 nm. No remarkable changes were observed in the MCF-7 cells and PBMCs toxicities with estrogenized HSA (4-OHE_2_-HSA) (**Figure 5**). 4-OHE_2_ and HSA alone served as controls. Thus, these results showed no direct effect of native or estrogenized HSA on the MCF-7 breast cancer cells or PBMCs.

### Detection of autoantibodies against estrogenized-HSA in breast cancer patients

**Figure 6:**
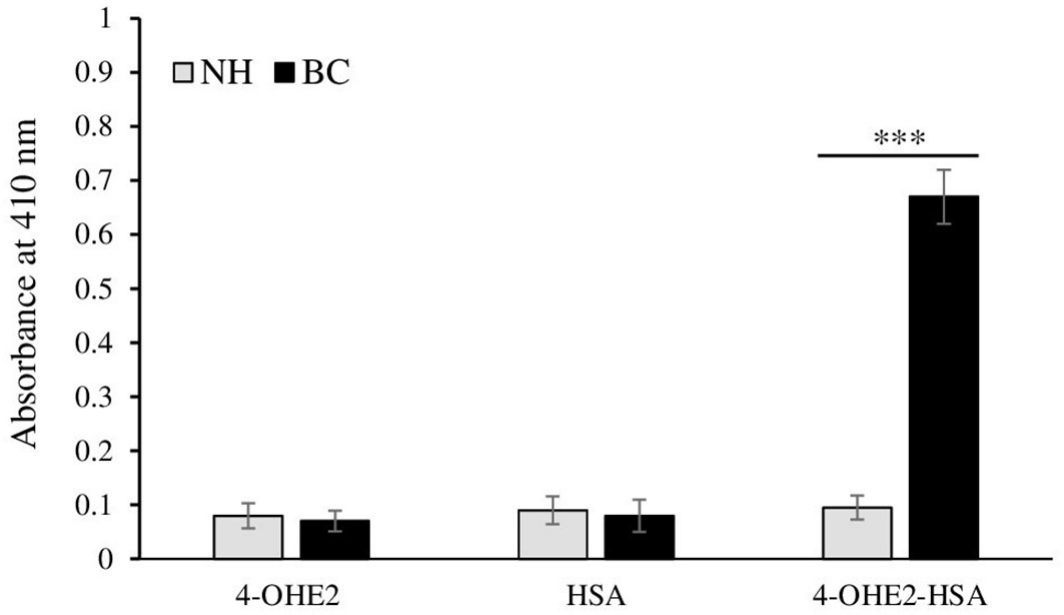
Direct binding ELISA of control (n = 45) and breast cancer (n = 85) patients’ antibodies 4-OHE_2_-HSA, HSA, and 4-OHE_2_. Data presented as mean ± SD (0.81 ± 0.23, median=0.58). *** represents *p*-value < 0.001 when breast cancer samples were compared with normal subjects.

To conduct an evaluation of the role of estrogenized HSA, ELISA was used to detect the antibodies against estrogenized HSA in different serum samples of BC patients. Data from direct binding ELISA showed a strong recognition of 4-OHE_2_-HSA (0.67 ± 0.051; optical density) by the serum antibodies in breast cancer patients as compared to healthy controls (*p* < 0.001) (**Figure 6**). HSA and 4-OHE_2_ did not show binding with patient sera antibodies. All three antigens (4-OHE_2_-HSA, HSA, and 4-OHE_2_) showed negligible binding with serum samples from healthy individuals. This result shows that 4-OHE_2_-HSA plays a significant role in antibody generation in breast cancer patients.

**Figure 7:**
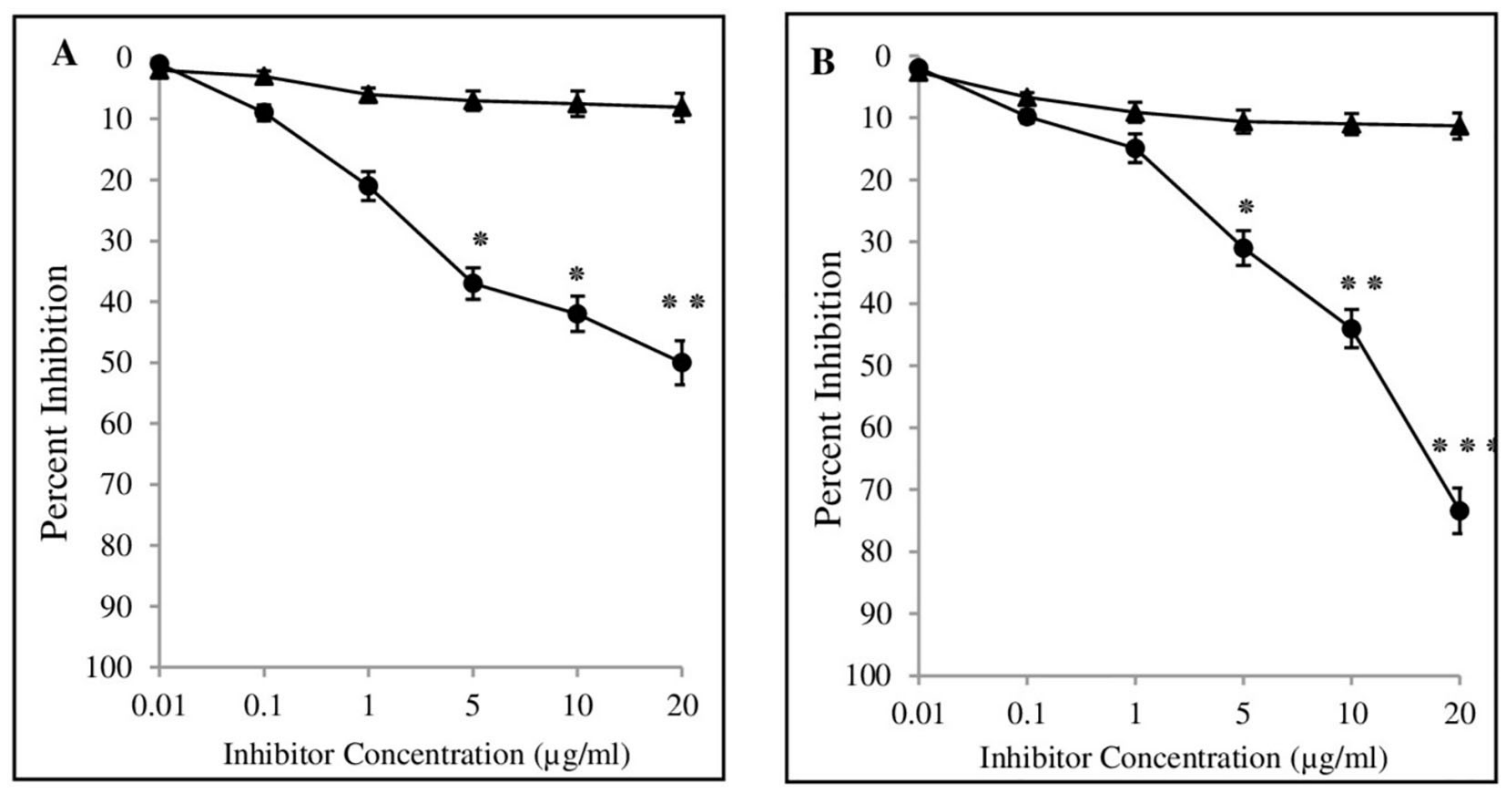
Inhibition ELISA of serum samples from cancer patients (-•-) and healthy controls (-▴-) were assessed against 4-OHE_2_-HSA **(A)**. Inhibition ELISA of isolated IgGs from serum samples of breast cancer patients (-•-) and healthy controls (-▴-) against 4-OHE_2_-HSA **(B)**. Microtitre plates were coated with respective antigens (2.5 μg/ml). *, **, and *** represent *p*-values < 0.05, < 0.01, and < 0.001.

The binding specificity of BC antibodies with 4-OHE_2_-HSA was detected by using inhibition ELISA. Estrogenized HSA showed an inhibition value of about 50.3 ± 4.6% (range 41.4% to 62.3%) in the sera of 85 breast cancer patients (**Figure 7A**). Significant differences (*p* < 0.05) were observed in the inhibition values between BC and NH sera samples at 5 µg/ml antigen concentration, and the difference was more pronounced (*p* < 0.01) at 20 µg/ml. IgGs isolated from the BC samples exhibited significantly higher levels (*p* < 0.001) of inhibition of 4-OHE_2_-HSA (73.4 ± 5.6) (**Figure 7B**) at 20 µg/ml. In contrast, sera samples and IgGs from healthy individuals demonstrated inhibition of 8.1 ± 2.3 and 11.3 ± 1.9% against 4-OHE_2_-HSA (**Figure 7B**).

The inhibition value was also determined for various groups/conditions in different BC patients (**Table 2**). BC patients were divided into four groups and inhibition values were determined accordingly. Cancer patients without treatment demonstrated the highest inhibition values (84.8 ± 5.2%), followed by patients with chemotherapy (79.8 ± 5.8%), positive estrogen receptor (76.4 ± 5.3%) and surgery + chemotherapy (71.4 ± 6.4%), etc. Inhibition values were independent of other parameters such as menopausal status, and smoking.

**Table 2:** Clinical and inhibition ELISA data of various BC patients.

	Inhibition (%) at a concentration of 20 μg/ml		
	4-OHE_2_-HSA	HSA	4-OHE_2_
Breast cancer patients (n=85)			
Estrogen receptor			
- Positive (n=61)	76.4±5.3	9.9±1.2	9.3±1.1
- Negative (n=24)	66.3±5.6	6.8±1.4	7.2±1.8
Smoking at baseline			
- Current/Past (n=45)	66.9±6.2	9.5±1.4	9.5±1.4
- Never (n=40)	59.7±4.9	8.4±1.6	6.2±1.2
Treatment			
- None (n=20)	84.8±5.2	8.8±1.7	9.4±1.2
- Surgery (n=15)	75.1±4.3	7.3±1.9	10.1±1.9
- Chemotherapy (n=23)	79.8±4.8	7.6±1.1	7.3±2.1
- Surgery + radiotherapy (n=10)	66.5±6.7	9.1±1.2	7.9±1.6
- Surgery + chemotherapy (n=17)	71.4±6.4	9.7±1.4	9.9±1.7
Menopausal status			
- Premenopausal (n=29)	68.8±5.9	7.8±2.1	7.1±1.2
- Postmenopausal (n=56)	79.3±5.1	8.7±1.5	7.5±1.4
Overall BC patients (n=85)	72.3±5.36	8.5±4.5	8.3±1.3
NH (n=45)	8.1±1.7	6.6±1.3	8.8±1.1

Values are given in mean±SD.

4-OHE_2_-HSA, HSA, and 4-OHE_2_ were used as inhibitors.

### Serum IgG affinity with 4-OHE_2_-HSA

**Figure 8:**
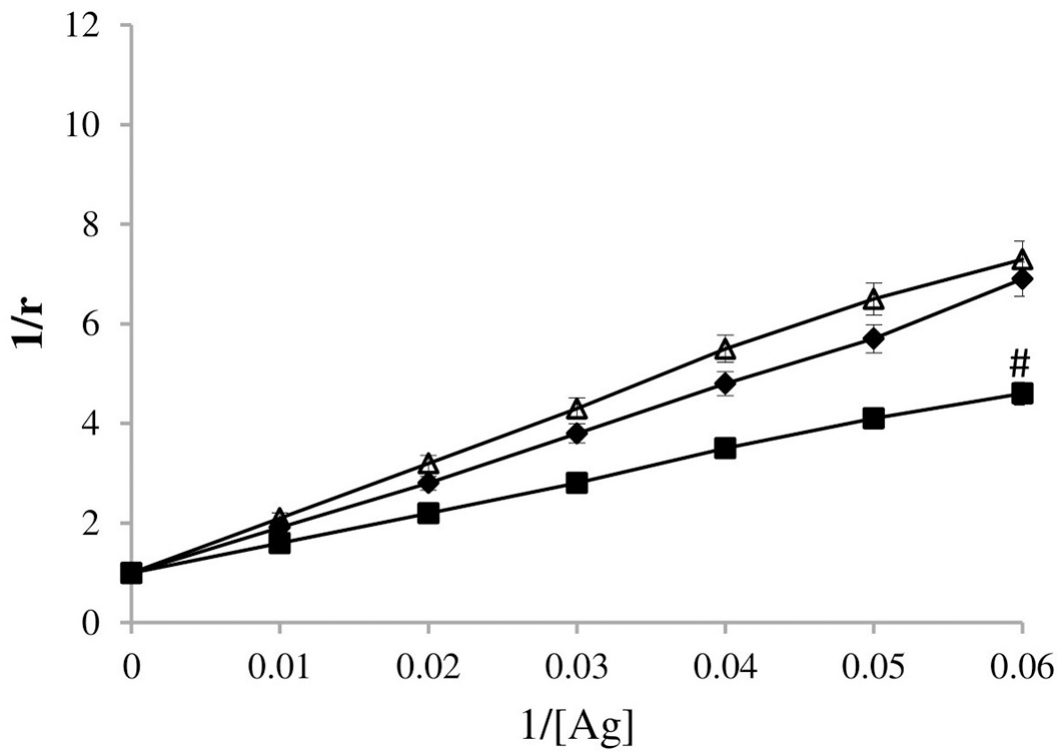
Estimation of an apparent association constant by Langmuir plot. Antigens used were 4-OHE_2_-ER (-▪-), HSA (-♦-) and 4-OHE_2_ (-Δ-). #represents *p* < 0.01. ‘r’ = maximum (saturation) surface binding of antigen by specific antibodies.

The binding between the antigen and serum IgG was also investigated using quantitative precipitation titration. Varying amounts of antigens (estrogenized HSA, HSA, 4-OHE_2_) were taken with a constant amount of purified breast cancer patient’s sera IgG. IgG from normal individuals served as controls. Precipitation titration experiment showed that about 26 μg of estrogenized HSA bound to 73 μg of BC patient’s sera IgGs. With HSA, 38 μg of antigen bound to 61 μg of BC patient’s sera IgGs. A maximum of 51 μg of 4-OHE_2_ bound to 59 μg of BC patient’s sera IgGs. The apparent association constant was calculated by determining the precipitation titration curve (Langmuir plot). Langmuir plot showed apparent association constants of 1.31 × 10^-7^ M, 1.68 × 10^-6^ M, and 1.36 10^-6^ M for 4-OHE_2_-HSA, HSA, and 4-OHE_2_, respectively (**Figure 8**). The constant clearly showed the maximum affinity of cancer IgG for 4-OHE_2_-HSA when compared with other antigens. 4-OHE_2_-ER antigen showed significantly (*p* < 0.01) higher binding with IgG than HSA and 4-OHE_2._

### Serum cytokines analysis

Cytokines IL-6 and IL-1 and TNF-α are considered pro-inflammatory cytokines and play a critical role in BC pathogenesis. BC patients showed higher levels of serum IL-1 (97.67 ± 18.36 pg/mL; p<0.05), IL-6 (33.42 ± 6.42 pg/mL; p<0.001) and TNF-α (102.15 ± 21.51 pg/mL; p<0.01) as compared to healthy controls (62.90 ± 13.33 pg/mL, 8.92 ± 1.58 pg/mL, and 39.91 ± 8.13 pg/mL, respectively) (**Table 2**).

## Discussion

Metabolism of sex hormones is altered in breast cancer patients and may exert different effects on mammary cells over a period of time. There is substantial evidence (29–31) for genotoxic, mutagenic and proliferative activities of different estrogen metabolites in breast cancer. These metabolites include 2-hydroxyestrogen, 4-hydroxyestrogen, and 16α-hydroxyestrogen, produced by a series of oxidizing enzymes of the cytochrome P_450_ family at positions 2, 4, and 16 carbon of the mother compound. Among them, 4-hydroxyestrogen is the most active metabolite, that alters the function of the spindle-assembly checkpoint and causes genomic instability (32).

4-hydroxyestrogen is a more potent carcinogen than 2-hydroxyestrogen and exhibits greater carcinogenic activity as compared to 2-hydroxyestrogen (33, 34). The lower dose of 4-OHE_2_ (70 nM) was found to be mutagenic once incubated with epithelial cells, whereas 2-OHE_2_ can cause mutation only at the higher dose (3.6 μM) (35). 4-hydroxyestrogen or its quinone derivatives were significantly elevated in the biopsies of breast tumor cells compared to normal cells (16). The enzyme cathechol-O-methyltransferase metabolizes 2-OHE_2_ at a faster rate compared to 4-OHE_2_. This shows greater likelihood of 2-methylation over 4-methylation and may explain the lowered tumorigenic potency of 2-hydroxy derivatives (36). Treatment of human breast cells with 4-OHE_2_ leads to the production of 8-oxo-7,8-dihydroxy-2’-deoxyguanosine. Furthermore, these catechol metabolites also generate OH radicals in breast epithelial cells (37). ROS produced by 4-hydroxyestradiol further stimulates the signaling of IkB kinase (IKK)-Nuclear factor kappa B (NF-κB) cascade, which in turn results in the transformation of normal cells to carcinogenic cells (38).

Previous studies have documented the presence of estrogenized proteins in human serum. These estrogen-protein adducts typically form through covalent bonding between estrogen metabolites and the cysteine, lysine, and histidine residues of proteins (9, 39). Such bonding can alter the protein structure, as demonstrated by biophysical analyses (UV and fluorescence spectroscopy) comparing 4-OHE_2_-HSA with native HSA. The cysteine-34 residue in HSA, the only free cysteine in the protein, is essential for antioxidant functions in the cytosol and transports various endogenous and exogenous ligands (9, 39). Consequently, the binding of 4-OHE_2_ to HSA may lead to structural and functional changes.

4-OHE_2_-HSA exhibited 33% UV hyperchromicity at 280 nm. 4-OHE_2_ forms covalent bonds with HSA, mainly through Cys, Lys, or His residues (9). Estrogenized HSA was tested for its binding proteins as well as the HSA receptor. However, no binding was observed between 4-OHE_2_-HSA and HSA binding proteins (cubilin, megalin) including FcRn, and IgG. This shows high likelihood that 4-OHE_2_ forms a stable protein adduct in the cells, playing an important role in BC pathogenesis. Moreover, no remarkable toxicities were observed in MCF-7 cells and human PBMCs when cocultured with estrogenized HSA or native HSA.

Increased levels of antibodies against 4-OHE_2_-HSA were detected in BC patients. Inhibition ELISA and quantitative precipitin titration assays were employed for the detection of binding specificities of the circulatory antibodies and antigen 4-OHE_2_-HSA. Data from both assays showed that circulatory antibodies recognized 4-OHE_2_-HSA to a greater extent. This explains how 4-OHE_2_-HSA might generate epitopes that lead to the production of circulatory antibodies in breast cancer. The present study has shown similar results to the earlier studies (11–13), demonstrating strong recognition of estrogenized protein in breast cancer and diabetes. This preference was not shown by HSA or 4-OHE_2_. The specificity of cancer antibodies was also tested for different groups/conditions associated with breast cancer patients. Cancer patients experiencing depression demonstrated (12) the highest inhibition values, followed by patients undergoing chemotherapy, those positive for estrogen receptor and those receiving surgery + chemotherapy. Depression has been shown to increase the production of antibodies against estrogenized protein in BC patients (31). It also increases antibodies in type I diabetes. Additionally, depression stimulates production of high-affinity autoantibodies against estrogenized protein in prostate cancer patients under inflammatory conditions (11). Substantial (76.4 ± 5.3%) binding of 4-OHE_2_-HSA with circulatory antibodies was observed in estrogen receptor-positive BC patients. About 70% of breast cancer tumors express estrogen receptors, and the drug tamoxifen, which binds to estrogen receptors, is the most used drug for BC (40). Thus, the hormone receptor is an important predictive and prognostic biomarker (41, 42). In another estrogen-related condition, endometriosis, estrogen receptor levels have been reported to increase more than 100-fold, accompanied by elevated levels of proinflammatory cytokines (43, 44). Similarly, inhibition ELISA data from BC patients with estrogen receptor-positive status show a significantly higher recognition of IgG antibodies (76.4 ± 5.3%). The presence of autoantibodies against ERs may enhance binding affinity, which, in the presence of 4-OHE_2_, could trigger heightened immunological responses. Immunological responses generated in patients with chemotherapy may lead to production of different types of autoantibodies. Chemotherapy is associated with numerous side effects, impacting both the physiological and psychological aspects of a patient’s life (45). Certain therapeutic interventions might suppress immune responses against cancer cells. Cyclophosphamide and methotrexate have been shown to impair the proliferative and effector functions of T cells (46, 47). Similarly, Imatinib mesylate suppresses the division of memory CTLs but has no effect on T or B cell responses (48). Gemcitabine exerts a selective effect on humoral immune responses (49). Evidence (47) suggests that therapy for cancer may benefit through the involvement of the immune system in two ways: firstly, by the stimulation of specific cellular responses that can immunogenically destroy tumor cells, and secondly, through the use of various chemical agents that might induce immunogenic death of tumor-cell. Other conditions, such as menopausal status, smoking, and disease duration, have no major effects on inhibition values of 4-OHE_2_-HSA with breast cancer antibodies. Their binding remains the same if compared with overall inhibition values in these patients.

The data from the affinity constant demonstrated high binding of 4-OHE_2_-HSA compared to HSA or 4-OHE_2_. Inhibition values of 4-OHE_2_-HSA by cancer autoantibodies clearly showed the presence of the adduct/epitopic regions involved in BC. It is important to note that inflammatory cytokines (IL-1, IL-6, and TNF-α) in BC patients were significantly higher as compared to controls. These cytokines participate in angiogenesis and play an executive role in cancer cell proliferation and metastasis by controlling NF-κB, IL-6/JAK/STAT3, PI3K-PKB/Akt, Ras/Raf, JNK, MAPK, and AKT pathways (50). They also modulate the tumor microenvironment by promoting the production of extracellular matrix components and stimulating the recruitment of immune cells. This is somewhat related to the proinflammatory state in these patients. Higher levels of pro-inflammatory cytokines such as IL-6 and TNF-α, in combination with other cytokines and factors may stimulate the production of antibodies via different pathways (51–53), which may facilitate stronger binding to the antigen. Increased secretion of proinflammatory cytokines correlate with the presence of elevated levels of antibodies in BC patients in our study.

**Figure 9:**
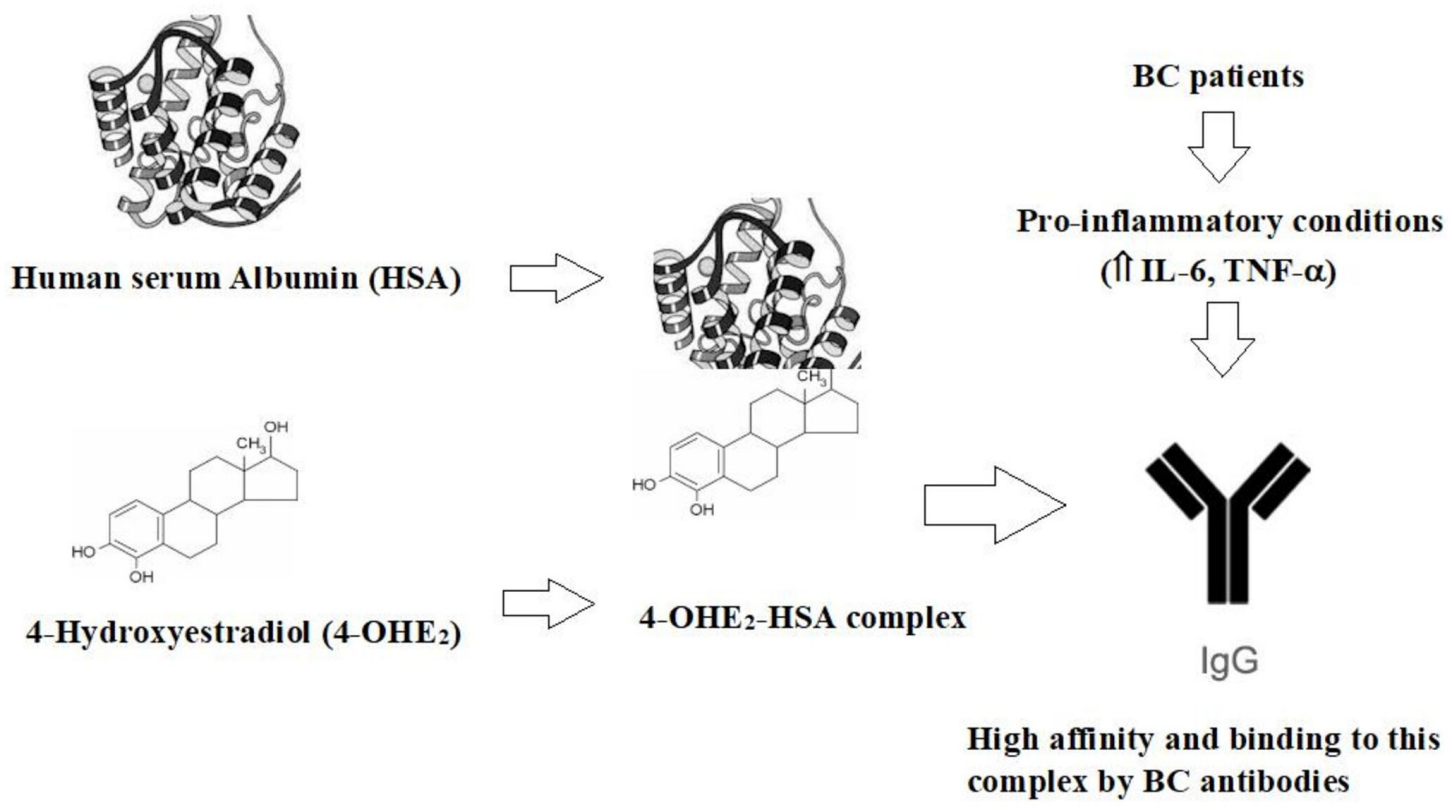
The proposed mechanism for the generation of autoantibodies (IgGs) in BC patients.

Data from this study clearly showed the antigenic role of 4-OHE_2_-HSA in BC patients. This study postulates that 4-OHE_2_ and HSA induced unique epitopes on estrogenized HSA that may be recognized by B-cells. This process may lead to the activation of B cells and differentiation into plasma cells that produce circulatory antibodies. Additionally, activated B cells may contribute to the production of pro-inflammatory cytokines (IL-1, IL-6, and TNF) -α (**Figure 9**). Thus, anti-4-OHE_2_-HSA antibodies can serve as an important immunological biomarker for the detection of BC.

The current study has some limitations that should be addressed in future research. Incorporating additional immunological assays, such as Western blotting for antigen and antibody recognition, as well as inflammatory cytokine analysis, could provide a more comprehensive understanding of the immune dysregulation associated with breast cancer pathogenesis. Furthermore, exploring the use of synthetic or biological molecules to inhibit the formation of adducts between HSA and 4-OHE_2_ would help clarify the role of 4-OHE_2_-HSA interactions in the development of BC. Future studies will aim to investigate these areas to address the remaining unanswered questions.

## Conclusions

The enhanced recognition of 4-OHE_2_-HSA by circulating antibodies in sera from breast cancer patients demonstrates the antigenicity of the molecule and suggests its potential role in triggering antibody production in these patients. One possible mechanism for the origin of BC antibodies may involve generation of a pro-inflammatory environment (increased IL-6, TNF-α). Future research should focus on developing monoclonal antibodies against 4-OHE2-HSA, which can be utilized to quantify the antigen in blood samples from a larger cohort of subjects. Given its high immunogenicity, 4-OHE2-HSA may be a valuable candidate for screening BC patients in clinical diagnostics. Additionally, screening for anti-4-OHE2-HSA antibodies in cancer patients could improve diagnostic and prognostic outcomes. These antibodies may serve as potential biomarkers for BC diagnosis and prognosis, and 4-OHE2-HSA itself could be explored as a molecular target for early disease detection.

## Data Availability

The original contributions presented in the study are included in the article/supplementary material. Further inquiries can be directed to the corresponding authors.
